# Therapeutic potential of urine exosomes derived from rats with diabetic kidney disease

**DOI:** 10.3389/fendo.2023.1157194

**Published:** 2023-05-12

**Authors:** Deendayal Das Mishra, Biswajit Sahoo, Pramod Kumar Maurya, Rajni Sharma, Santosh Varughese, Narayan Prasad, Swasti Tiwari

**Affiliations:** ^1^ Department of Molecular Medicine & Biotechnology, Sanjay Gandhi Postgraduate Institute of Medical Sciences, Lucknow, India; ^2^ Department of Nephrology, Christian Medical College, Vellore, India; ^3^ Department of Nephrology, Sanjay Gandhi Postgraduate Institute of Medical Sciences, Lucknow, India

**Keywords:** urinary exosomes, kidney biopsy, diabetic nephropathy, renal fibrosis, therapeutics

## Abstract

Kidney disease is prevalent in diabetes. Urinary exosomes (uE) from animal models and patients with Diabetic nephropathy (DN) showed increased levels of miRs with reno-protective potential. We examined whether urinary loss of such miRs is associated with their reduced renal levels in DN patients. We also tested whether injecting uE can leverage kidney disease in rats. In this study (study-1) we performed microarray profiling of miRNA in uE and renal tissues in DN patients and subjects with diabetes without DN (controls). In study-2, diabetes was induced in Wistar rats by Streptozotocin (i.p. 50 mg/kg of body weight). Urinary exosomes were collected at 6th, 7th and 8th weeks, and injected back into the rats (100ug/biweekly, uE-treated n=7) via tail vein on weeks 9 and 10. Equal volume of vehicle was injected in controls (vehicle, n=7). uE from the human and rat showed the presence of exosome-specific proteins by immunoblotting. Microarray profiling revealed a set of 15 miRs having high levels in the uE, while lower in renal biopsies, from DN, compared to controls (n=5-9/group). Bioinformatic analysis also confirmed the Renoprotective potential of these miRs. Taqman qPCR confirmed the opposite regulation of miR-200c-3p and miR-24-3p in paired uE and renal biopsy samples from DN patients (n=15), relative to non-DN controls. A rise in 28 miRs levels, including miR-200c-3p, miR-24-3p, miR-30a-3p and miR-23a-3p were observed in the uE of DN rats, collected between 6th-8th weeks, relative to baseline (before diabetes induction). uE- treated DN rats had significantly reduced urine albumin-to-creatinine ratio, attenuated renal pathology, and lower miR-24-3p target fibrotic/inflammatory genes (TGF-beta, and Collagen IV), relative to vehicle treated DN rats. In uE treated rats, the renal expression of miR-24-3p, miR-30a-3p, let-7a-5p and miR-23a-3p was increased, relative to vehicle control. Patients with diabetic nephropathy had reduced renal levels, while higher uE abundance of miRs with reno-protective potential. Reverting the urinary loss of miRs by injecting uE attenuated renal pathology in diabetic rats.

## Introduction

Diabetic nephropathy (DN) is one of the severe complications of diabetes (1, 2). MicroRNAs (miRNAs/miRs) may be essential in the onset and progression of diabetic nephropathy (3, 4). We and others have demonstrated that inhibiting certain miRs, crucial for kidney health promoted fibrosis in rats (5–7). MicroRNA are small, endogenous RNA molecules ~20–24 nucleotides in length. They play a significant role in post-transcriptional gene regulation via transcript inhibition or destabilization by base pairing to their target sequence motifs in the 3′ UTR of mRNA (8–13). Thus, an aberration in the expression of miRNA affects biological processes by regulating genes crucial to disease initiation or progression. We and others have reported microRNA dysregulation in the kidney tissue in DN that may be causally associated with the occurrence and development of renal fibrosis (14–17). For example, the DN-associated decline in the expression of renoprotective miRs in kidney tissue may further promote fibrosis (18). However, the possible mechanism of changes in the miR expression in kidney tissue is unclear.

Besides regulation of miR synthesis, increased secretion of cellular miRs in the extracellular space could be a possible mechanism (19). Cell-free miRNAs are either associated with high-density lipoproteins and RNA-binding argonaute proteins or enclosed within extracellular vesicles (EVs) (20–22). EVs are naturally secreted by every cell into the extracellular space; they can be divided into three categories based on their biogenesis, size, pathways, function, and content, microvesicles (MVs), exosomes, and apoptotic bodies. Kidney cell-derived exosomes that appear in urine (uE) are released by the fusion of multivesicular endosomes with the apical membrane of renal epithelial cells. The uE cargoes may reflect the renal response to pathological/physiological stimuli. It has been suggested that exosomes contain a defined set of a biomolecule, including miRNAs, through precisely regulated pathways that depend upon the status of the cells from which it was derived (23, 24). However, a few studies, including ours, demonstrated that the regulation of certain miRs in exosomes might differ from that of the cell of origin (22, 25, 26). Recently, Ruben et al., 2021 have shown the presence of cell-specific motifs on a few miRNAs that could force their cell retention. The random movement of miRNAs from cell cytoplasm into the multivesicular bodies can also happen (27). Moreover, through cell-to-cell communication, exosomes could profoundly regulate recipient cell transcriptome via lateral transfer of their miRNAs content (28).

Nevertheless, how miRs regulation in uE relates to kidney tissue may be of immense significance in human DN. For example, urinary loss of renoprotective miRs through urinary exosomes could reduce their expression in kidney tissue and thus affect disease pathogenesis/progression. Recouping the loss of such miRs may leverage kidney disease therapeutics. We identified DN-associated miRs in humans that show higher expression in uE but lower in renal biopsy tissue. In rats with DN, we tested whether injecting back the lost miRs through uE injection could recover their renal levels and attenuate pathology.

## Materials and methods

### Human participants (study 1)

Subjects with type 2 diabetes mellitus (T2DM) with or without kidney disease, and their age-matched respective controls were enrolled after written consent (**Tables 1**, **2**). The study was approved by the institutional Ethics committee (IEC) of the Sanjay Gandhi Postgraduate Institute of Medical Sciences (IEC Code 2018-139-EMP-106). Patients suffering with urological abnormalities, recurrent urinary tract infection, HIV, Hepatitis B/C, nephrolithiasis, underlying heart disease (angina class 3 or above) or having acute changes in serum creatinine (Scr) of more than 20% within the past 4 months were excluded. Vulnerable subjects like pregnant women and children were also excluded. Besides, individuals having an inability or unwillingness to provide written consent or had organ transplantation, or history of malignancy (past or current) were excluded.

**Table 1:** The Fold change in the expression of the miRs that showed opposite regulation in urinary exosomes (uE) and renal biopsies from T2DM patients with diabetic nephropathy (DN), relative to respective controls.

		Urinary exosomes (uE)	Renal biopsy tissues
S.No	Transcript ID	Fold change (DN vs. DC)	Fold change (DN vs. Cnt.)
1	hsa-miR-103a-3p	4.62	-5.36
2	hsa-miR-151a-5p	3.25	-4.6
3	hsa-miR-191-5p	8.51	-6.35
4	hsa-miR-1972	6.89	-3.18
5	hsa-miR-22-3p	3.78	-32.05
6	hsa-miR-24-3p	5.87	-7.06
7	hsa-miR-26a-5p	10.09	-8.1
8	hsa-miR-30d-5p	2.32	-10.37
9	hsa-miR-361-5p	2.77	-4.1
10	hsa-miR-378a-3p	3.13	-4.04
11	hsa-miR-4454	10.01	-2.37
12	hsa-miR-200c-3p	40.43	1.07
13	hsa-miR-619-5p	29.01	-6.83
14	hsa-let-7i-5p	2	-4.72
15	hsa-miR-574-3p	2.16	-2.66

The miRs expression were higher in uE from DN patients, relative to age matched T2DM patients without kidney disease (DC, n=9/group). However, in renal biopsies from DN patients (n=5) the fold change in the expression was lower relative to renal biopsies collected from patients undergoing nephrectomy for renal calculus were taken as controls (Cnt, n=3). The microarray data was analyzed using TAC 4.0 analysis software.

Renal biopsies were collected from T2DM subjects with kidney disease who underwent kidney biopsies in the department of Nephrology at Sanjay Gandhi Postgraduate Institute of Medical Sciences, Lucknow, and Christian Medical College, Vellore. Tissues collected from the normal region of the kidney cortex of age-matched subjects undergoing nephrectomy for renal calculus served as controls. Besides, urine samples were collected from T2DM patients with non-diabetic kidney disease (Disease controls, n=6). Samples were stored at -80°C for further analysis. A second-morning urine samples were collected from all the enrolled participants. Urine samples were subjected to low spin centrifugation (1000 × g 10 min) to remove cellular debris and stored at −80°C for exosome isolation.

### Animal study (study 2)

The animal study was approved by the Institutional Animal Ethics Committee (IAEC) of Sanjay Gandhi Postgraduate Institute of Medical Sciences (Ref no. IAEC/P-21/25/2018), according to guidelines of Control and Supervision on Experiments on Animals (CPCSEA), Ministry of Environment and Forests, Government of India. Male Wistar rats (weight 230–280 g, age 90–100 days) were obtained from our in-house Animal facility. Animals were acclimatized under laboratory conditions for 2 weeks, housed in standard rat cages with 3 rats in each cage at 23–25°C and humidity-(50–60%) controlled condition with a 12-hour light/dark cycle. Animals had free access to a standard chow diet and tap water. Diabetes mellitus was induced in (n=16) rats via single dose of intraperitoneal (I.P) administration of streptozotocin (STZ) (50mg/kg body weight) dissolved in 0.1M citrate buffer as described previously by us (14). Blood glucose levels were monitored from the tail vein using a glucometer (Abbott Freestyle Optium NEO H Glucometer, Abbott Diabetes Care Inc. Alameda, CA, USA). Rats were considered diabetic when their blood glucose exceeded 200 mg/dL (11 mmol/L). Urinary exosomes (uExo) were collected from DM rats at 6th, 7th, and 8th weeks, and pooled. At 9^th^ and 10^th^ week of diabetes inductions, the DM rats were either injected with an aliquot of the pooled uExo, 100ug/biweekly (treated; n=9) or an equal volume of vehicles (Vehicle, n=7). The treatment was given via tail vein injection. Rats were euthanized after 2 weeks of treatment under Isoflurane 2% anesthesia (Sigma Chemical Co., St. Louis, MO, USA). Blood was collected through cardiac puncture. Rat kidneys were collected after perfusion with 1X phosphate-buffered saline (PBS). The left kidneys were kept in 4% paraformaldehyde for histological analysis whereas the right kidneys were stored at -80°C for RNA isolation.

### Urine analysis

Urine albumin level was evaluated using a rat albumin ELISA kit (Bethyl Laboratories, TX, USA) and urine creatinine level was assessed using a modified Jaffe’s method (Randox, Crumlin County Antrium, UK).

### Isolation and characterization of urinary exosomes

Exosomes were isolated from human and rat urine samples by differential ultracentrifugation as previously described (14, 29, 30). Size distribution and concentration of exosomes were analyzed by Nanoparticle Tracking Analysis (NTA) using the NanoSight NS300 (Malvern Instruments Ltd., UK) at the Central Analytical Research Facility of the Indian Institute of Toxicological Research, Lucknow, according to the manufacturer’s protocol.

### Immunoblotting

Exosomal proteins were subjected to immunoblotting as previously described (14, 30). For immunoblotting, antibodies against CD9 (Clone ab92726), CD63 (Clone ab59479), CD81 (Clone ab23505), and TSG101 (Clone ab83) (Abcam, Cambridge, UK) were used at a 1:500 dilution followed by incubation with HRP conjugated secondary antibodies at (1:1000) dilutions (Abcam, Cambridge, UK). All the images were acquired using ChemiDoc Imaging System (Universal Hood III, BIO-RAD, CA, USA).

### RNA isolation

Total RNA from renal tissue was extracted using Quizol Reagent (Quigen, Hilden, Germany). Briefly, 700μL qiazol was used per 100 mg tissue. The tissue was homogenized and incubated at RT for 5 minutes followed by chloroform (200 μl) addition. The samples were vortexed for 15 seconds and incubated for 3 minutes at RT. Samples were then centrifuged at 12,000 x g for 15 min at 4°C and the aqueous phase was transferred to a fresh tube. RNA was precipitated by adding 500μl isopropanol and the pellet was collected after centrifugation at 12,000 x g for 10 minutes at 4°C. The RNA pellet was washed by vortexing with 75% ethanol and subsequently centrifuged at 7,500 x g for 5 minutes at 4°C. The RNA was dissolved in RNase-free water (Invitrogen, MA, USA). RNA quality and quantity were determined by a Spectrophotometer (NanoDrop™ 2000/2000c).

### Microarray analysis

miRNA expression profiling was done by Affymetrix miRNA 4.0 Array (Affymetrix- GeneChip™ miRNA 4.0 Array; Thermo Fisher Scientific, Inc., Waltham, MA, USA). Biotin-labeled RNA was prepared from an Affymetrix^®^ FlashTag TM Biotin HSR RNA Labelling Kit (Thermo Fisher Scientific). In brief, ∼120 ng of total RNA was subjected to poly-A tailing at the 3’-end, followed by linking of biotin-labeled 3DNA molecule to the 3′-end by a DNA ligase. Thereafter, the biotin-labeled RNA samples were hybridized to GeneChip miRNA 4.0 arrays in an Affymetrix^®^ Oven 455 (Thermo Fisher Scientific) for 16-18 h at 48°C with 60 rpm rotation. Following hybridization, miRNA 4.0 arrays were subjected to washing and staining using the GeneChip^®^ Hybridisation, Wash, and Stain Kit (Thermo Fisher Scientific) and Fluidics Station 450 (Thermo Fisher Scientific). Hybridized targets on the array were stained with streptavidin–phycoerythrin provided in the kit and detected using Scanner 3000 7G (Thermo Fisher Scientific). The raw expression data in the form of.CEL file was analyzed using Affymetrix Transcriptome analysis console software (TAC 4.0, Thermo Fischer Scientific). After data normalization, the differential miRs expressions were obtained for both uE and renal samples.

### Real time qPCR

Real-time PCR (qPCR) validation of miRNAs was performed by using cDNA prepared from 10 ng of total RNA, enriched from uE, using commercially available Taqman micro-RNA expression assays (Applied Biosystems, Foster City, CA, USA) as per the manufacturer’s instructions. For mRNA, high-capacity cDNA synthesis kit (applied biosystems) was used for cDNA conversion according to the manufacture’s instruction. Takara TB Green Premix Ex Taq probes were used for relative quantification as per manufacture’s instruction. qPCR was performed using the BIO-RAD CFX96 Touch Real-Time PCR Detection System. All the reactions were performed in duplicates. Data were analyzed using the 2^-ΔCt^ method.

### Histopathology of renal tissues

Rat kidney tissues, fixed in 4% paraformaldehyde were processed in paraffin, sectioned at 3μm and stained with Periodic acid-Schiff (PAS) and Masson Trichrome (MT) according to the manufacturer’s instructions (Sigma, St Louis, MO) as described by us previously (14, 31). Stained sections were used for the following analysis using an Olympus IX73 light microscope.

### Functional annotation of miRNA

The functional enrichment analysis of identified miRNA signatures has been performed using miRNA 2.0 (miRNA Enrichment Analysis and Annotation Tool; https://www.ccb.uni-saarland.de/mieaa2) which provide the annotation of miRNAs in different categories such as pathway, disease, chromosomal location, site of expression, miRNA-TF interactions, family and PubMed annotation (32).

### Statistical analysis

Quantitative data are expressed as mean ± SEM. Comparisons within groups were made using paired Student t-tests. One-way ANOVA followed by multiple comparisons testing was used to assess differences between individual pairs of means among the groups. P values < 0.05 were considered significant for all tests using Sigma Plot 12.3 (Chicago, IL). Fold change in expression by RT-PCR were calculated by 2^-ΔCt^ method, where ΔCt = Ct_gene of interest_−Ct _endogenous control_.

## Results

### Characterization of urinary exosomes

**Figure 1:**
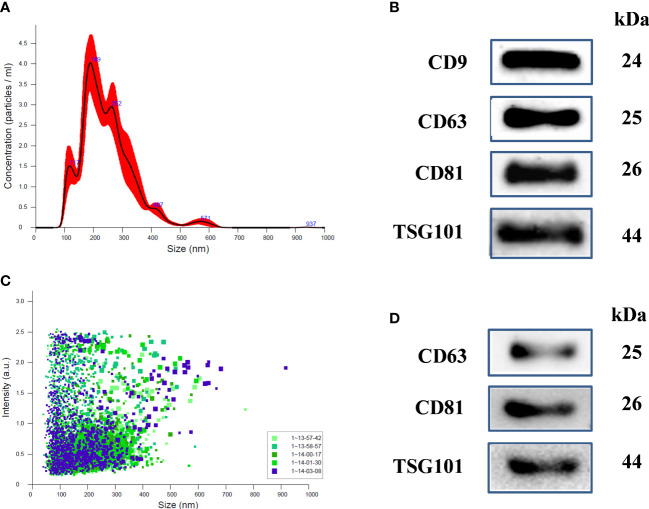
Characterization of uEs in human and Rats: **(A)** Nanoparticle tracking analysis (NTA) of uE showing exosome concentration (particles/mL)/size in pellets, **(B)** scattering distribution (intensity/size) profile, and **(C)** representative immunoblots showing the presence of exosome specific marker proteins, CD9, CD63, CD81, and TSG101 in the urinary exosomal (uE) protein of human samples. **(D)** representative immunoblots showing the presence of exosome specific markers, CD63, CD81, and TSG101 in rats. uE were isolated from human and rat urine samples via differential ultracentrifugation methods.

Physical characterization of human uE was done using NTA (Nanoparticle Tracking Analysis). **Figure 1** shows representative NTA plots of the average concentration (particles/mL), and size of vesicles isolated from human urine samples. The major peaks sizes at 117 nm, 189, and 262 nm in **Figure 1A** corresponding to exosomes. The concentration distribution clearly shows that the small size vehicles (>200nm) were much more abundant in our preparation than moderate or large size particles. Additionally, vesicles ranging from 40 to 200nm in size had higher intensity (**Figure 1B**).

Immunoblotting was also performed to examine the presence of exosome-specific marker proteins in humans (**Figure 1C**) and rat model (**Figure 1D**). We used 40 µg of uE protein for immunoblotting. The blots show the presence of specific protein bands for exosome-specific marker proteins; CD9, CD63, CD81, and TSG101.

### Loss of miRs with renoprotective potential through uE in human DN

**Figure 2:**
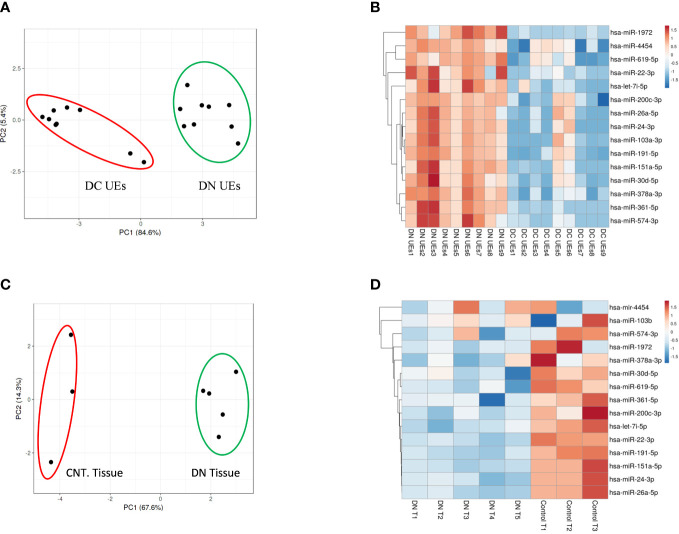
MicroRNA profiling of urinary exosomes (uE) and renal biopsy samples from patients with Diabetic nephropathy (DN). Principal Component Analysis plot, PCA **(A)** and Heatmaps, showing 15 up-regulated miRs from urinary exosomes **(B)** from DN patients, relative to age matched T2DM patents without kidney disease (DC, n=9/group); and renal biopsy samples (n=5, **(C, D)**, respectively) from DN patients, relative to age matched controls without chronic kidney disease (Cnt, n=3). The data was obtained using microarray analysis.

MicroRNA analysis of the renal biopsies and uE samples collected from the enrolled subjects were performed. **Supplementary Table S1** provides the detail of demographics of the enrolled participants. Microarray results showed, significant upregulation of 109 miRs (|fold change| ≥ 2, p-value < 0.05) in the urinary exosome of patients with diabetic nephropathy (DN), relative to T2DM patients without kidney disease (n=9/group) and those with non-diabetic kidney disease (NDKD, Disease controls, n=6).However, 15 (out of 109) miRs were upregulated in uE (**Figures 2A, B** **)**, and found downregulated in kidney tissue in microarray analysis, relative to controls (n=3-6/group, **Figures 2C, D**). Fold expression of these 15 miRs in uEs and kidney tissue are listed in **Table 1**, and their functional enrichment analysis is shown in **Table 2**. The database miEAA 2.0 showed renal expression of 11 of the 15 miRs, among which miR-24-3p and miR-200c-3p were highly prevalent in renal diseases, pathways and biological processes. These were validated and further analyzed.

**Table 2:** Functional enrichment and annotation analysis.

Functional annotation	Enriched functions	P-value	Observed	miRNAs/precursors
Diseases (MNDR)	type 2 diabetes mellitus	0.001218	4	hsa-miR-103b; hsa-miR-191-5p; **hsa-miR-24-3p**; hsa-miR-200c-3p
Diseases (MNDR)	Diabetic Nephropathies	0.00817	3	hsa-miR-24-3p; hsa-miR-26a-5p; **hsa-miR-200c-3p**
Diseases (MNDR)	diabetes mellitus	0.011561	8	hsa-miR-151a-5p; hsa-miR-22-3p; **hsa-miR-24-3p**; hsa-miR-26a-5p; hsa-miR-30d-5p; hsa-miR-361-5p; **hsa-miR-200c-3p**; hsa-miR-574-3p
Expressed in tissue (Tissue Atlas)	Kidney	0.014937	11	hsa-miR-151a-5p; hsa-miR-1972; hsa-miR-22-3p; hsa-miR-24-3p; hsa-miR-26a-5p; hsa-miR-30d-5p; hsa-miR-361-5p; hsa-miR-378a-3p; hsa-miR-200c-3p; hsa-let-7i-5p; hsa-miR-574-3p
GO Biological process (miRPathDB)	fibroblast proliferation	2.25e-5	5	hsa-miR-22-3p; hsa-miR-24-3p; hsa-miR-26a-5p; hsa-miR-200c-3p; hsa-miR-574-3p
GO Biological process (miRPathDB)	positive regulation of fibroblast proliferation	1.18e-5	5	hsa-miR-22-3p; hsa-miR-24-3p; hsa-miR-26a-5p; hsa-miR-200c-3p; hsa-miR-574-3p
GO Biological process (miRPathDB)	regulation of fibroblast proliferation	2.25e-5	5	hsa-miR-22-3p; hsa-miR-24-3p; hsa-miR-26a-5p; hsa-miR-200c-3p; hsa-miR-574-3p
GO Biological process (miRPathDB)	regulation of transforming growth factor beta receptor signaling pathway	1.82e-4	4	hsa-miR-22-3p; hsa-miR-26a-5p; hsa-miR-200c-3p; hsa-miR-574-3p
GO Biological process (miRPathDB)	positive regulation of gluconeogenesis	5.38e-4	2	hsa-miR-22-3p; hsa-miR-200c-3p
GO Biological process (miRPathDB)	positive regulation of protein kinase activity	5.07e-4	5	hsa-miR-22-3p; hsa-miR-24-3p; hsa-miR-26a-5p; hsa-miR-378a-3p; hsa-miR-200c-3p
GO Biological process (miRPathDB)	positive regulation of cell migration by vascular endothelial growth factor signaling pathway	8.92e-4	2	hsa-miR-378a-3p; hsa-miR-200c-3p
GO Biological process (miRPathDB)	renal tubule morphogenesis	0.001014	3	hsa-miR-378a-3p; hsa-miR-200c-3p; hsa-miR-574-3p
GO Biological process (miRPathDB)	nitrogen compound metabolic process	0.001061	7	hsa-miR-22-3p; hsa-miR-24-3p; hsa-miR-26a-5p; hsa-miR-30d-5p; hsa-miR-361-5p; hsa-miR-378a-3p; hsa-miR-200c-3p
GO Biological process (miRPathDB)	nephron morphogenesis	0.00116	3	hsa-miR-378a-3p; hsa-miR-200c-3p; hsa-miR-574-3p
GO Biological process (miRPathDB)	response to insulin	0.001319	3	hsa-miR-22-3p; hsa-miR-26a-5p; hsa-miR-200c-3p
GO Biological process (miRPathDB)	signal transduction involved in DNA damage checkpoint	0.001269	4	hsa-miR-24-3p; hsa-miR-26a-5p; hsa-miR-200c-3p; hsa-miR-574-3p
GO Biological process (miRPathDB)	extrinsic apoptotic signaling pathway	0.001561	4	hsa-miR-22-3p; hsa-miR-24-3p; hsa-miR-26a-5p; hsa-miR-200c-3p
GO Biological process (miRPathDB)	response to growth factor	0.002298	5	hsa-miR-22-3p; hsa-miR-26a-5p; hsa-miR-378a-3p; hsa-miR-200c-3p; hsa-miR-574-3p
GO Biological process (miRPathDB)	Gluconeogenesis	0.003142	2	hsa-miR-22-3p; hsa-miR-200c-3p
GO Biological process (miRPathDB)	regulation of apoptotic process	0.003842	5	hsa-miR-22-3p; hsa-miR-24-3p; hsa-miR-26a-5p; hsa-miR-378a-3p; hsa-miR-200c-3p
GO Biological process (miRPathDB)	negative regulation of epithelial cell apoptotic process	0.004749	2	hsa-miR-24-3p; hsa-miR-200c-3p
GO Biological process (miRPathDB)	negative regulation of endothelial cell apoptotic process	0.005669	2	hsa-miR-24-3p; hsa-miR-200c-3p
GO Biological process (miRPathDB)	transforming growth factor beta receptor signaling pathway	0.006793	3	hsa-miR-26a-5p; hsa-miR-200c-3p; hsa-miR-574-3p
GO Biological process (miRPathDB)	negative regulation of cellular response to insulin stimulus	0.01003	1	hsa-miR-200c-3p
GO Biological process (miRPathDB)	negative regulation of gluconeogenesis	0.01003	1	hsa-miR-200c-3p
GO Biological process (miRPathDB)	positive regulation of vitamin D biosynthetic process	0.01003	1	hsa-miR-24-3p
GO Biological process (miRPathDB)	activation of protein kinase activity	0.01239	3	hsa-miR-24-3p; hsa-miR-378a-3p; hsa-miR-200c-3p
GO Biological process (miRPathDB)	regulation of kinase activity	0.013831	4	hsa-miR-22-3p; hsa-miR-24-3p; hsa-miR-378a-3p; hsa-miR-200c-3p
Pathway (miRPathDB)	Wnt signaling pathway	4.36e-4	5	hsa-miR-151a-5p; hsa-miR-22-3p; hsa-miR-26a-5p; hsa-miR-200c-3p; hsa-miR-574-3p
Pathway (miRPathDB)	TGF-beta signaling pathway	0.005433	4	hsa-miR-22-3p; hsa-miR-378a-3p; hsa-miR-200c-3p; hsa-miR-574-3p
Pathways (KEGG)	Type I diabetes mellitus	0.001892	8	hsa-miR-103b; hsa-miR-191-5p; hsa-miR-1972; hsa-miR-24-3p; hsa-miR-26a-5p; hsa-miR-30d-5p; hsa-miR-619-5p; hsa-let-7i-5p
Pathway (miRWalk)	Apoptosis signaling pathway	8.32e-4	9	hsa-miR-151a-5p; hsa-miR-191-5p; hsa-miR-22-3p; hsa-miR-24-3p; hsa-miR-26a-5p; hsa-miR-30d-5p; hsa-miR-361-5p; hsa-miR-378a-3p; hsa-miR-200c-3p

Table detailed the results from the analysis including, pathways, gene ontology, expression sites and disease annotations of the miRs using miEAA 2.0 miRNA Enrichment Analysis and Annotation tool. P-value is calculated as adjusted p-value or FDR by using Benjamini–Hochberg method.

**Figure 3:**
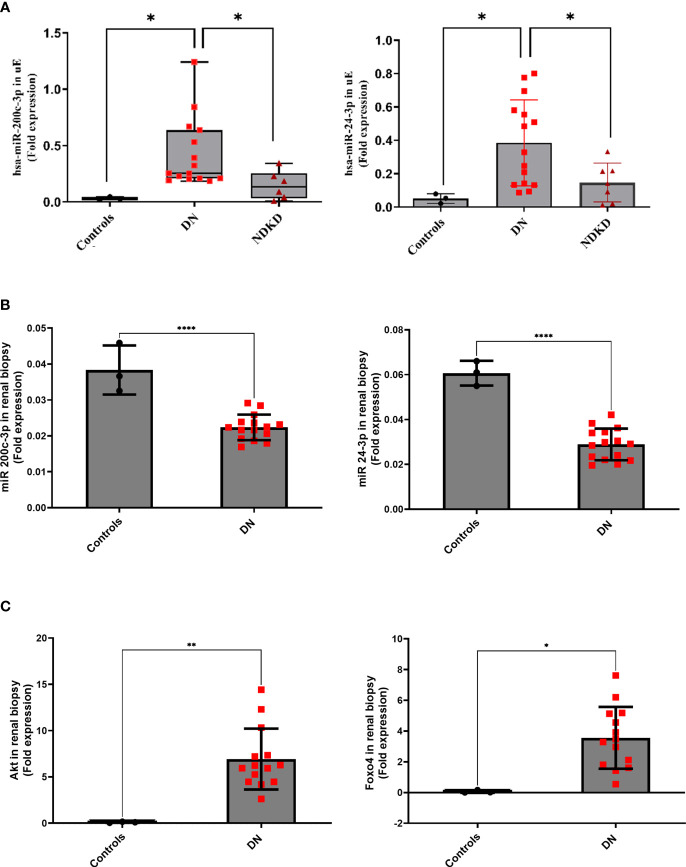
MicroRNA in paired uE and renal biopsy samples, and their target genes (in renal biopsy) from Diabetic nephropathy patients. Scatter dot plot with line at mean with SD showing fold expression of miR-200c-3p and miR-24-3p in paired uE **(A)** and renal biopsy samples from DN patients (n=15) **(B)**. Target genes AKT3 and FOXO4 **(C)** were analyzed in renal biopsies from DN patients. For comparisons, paired uE and renal biopsy from patients undergoing nephrectomy for renal calculus were taken as controls (n=3). NDKD uE were taken as disease control. The analysis was done by qPCR. 18S rRNA was used as the endogenous control. **p< 0.01 was considered as significant by unpaired t test. *p< 0.05 and ****p< 0.0001 were considered as significant.

The opposite regulation of miR-200c-3p, and miR-24-3p in uE and kidney tissue from DN patients (relative to controls) were confirmed by qPCR in paired uE and renal biopsy samples from DN patients (**Figures 3A, B**). **Supplementary Table S2** provides the detail of demographics of the participants from whom samples were collected. The fold expression of both the miRs in DN uE were significantly higher, relative to disease as well as non-disease controls (**Figure 3A**). The fold expressions were, however, significantly lower in the renal tissue from same DN patients, relative to non-disease control tissues (**Figure 3B**). The downregulation of these miRs in the kidney tissue corroborated with significantly higher expression of their targets, AKT3 and FOXO4 in the renal biopsy tissues from DN patients by qPCR analysis (**Figure 3C**).

**Figure 4:**
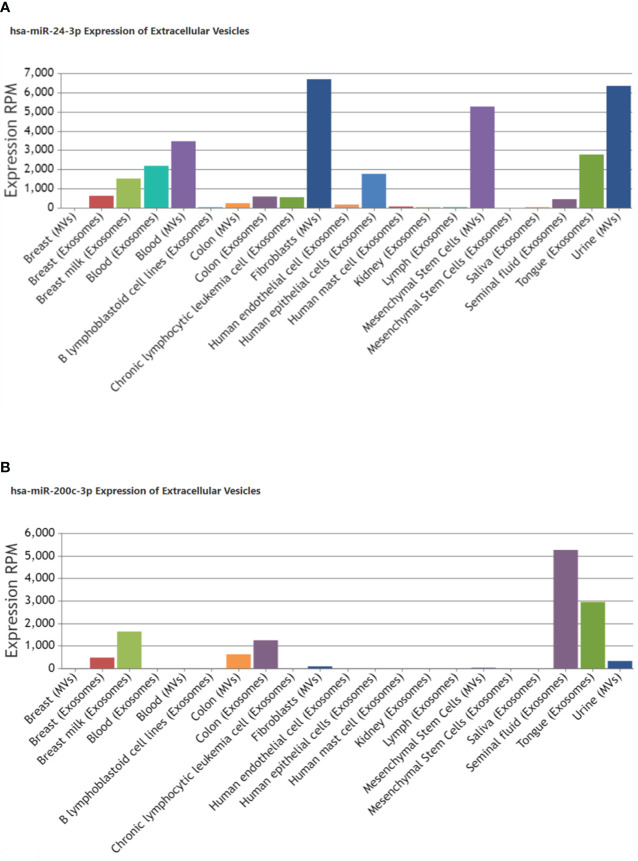
Localization of miR-24-3p and miR-200c-3p in extracellular vesicles derived from other tissue types in humans. Bar graph shows an expression of **(A)** hsa-miR-24-3p **(B)** hsa-miR-200c-3p reported in exosomes/microvesicles originated from different tissues or biofluids under pathological or physiological conditions. The Insilco analysis was done using EVmiRNA tool. The Y-axis indicates the expression levels, RPM, Reads Per Million.

Enrichment Analysis and Annotation also confirmed their valid and stable expression in kidney tissues along with their presence or localization in exosomes (**Table 2**; **Figure 4**). miR-24-3p and miR-200c-3p were associated with type 2 diabetes mellitus and Diabetic Nephropathies and were found to positively regulate processes such as fibroblast proliferation, protein kinase activity, nitrogen compound metabolic process, signal transduction involved in DNA damage checkpoint, extrinsic apoptotic signaling pathway. hsa-miR-200c-3p was found to stimulate Wnt, VEGF and TGF-beta signaling pathway, and suggested as a negative regulator for the cellular responses to insulin stimulus including gluconeogenesis. Compared to miR-200c-3p, the abundance of miR-24-3p was found substantially higher in the exosomes derived from fibroblasts and urine, using Extracellular Vesicles miRNA Database (EVmiRNA) tool (**Figure 4**). Therefore, we further focused on miR-24-3p and identified their target genes. A total of 3248 targets of miR-24-3p were identified, among which 108 targets were associated with lipolysis, FOXO signaling, autophagy, insulin resistance, VEGF signaling, growth hormone synthesis, secretion and action, and AGE-RAGE signaling pathways.

### uE from rats and patients with DN showed increased levels of similar miRs with reno-protective potential

**Figure 5:**
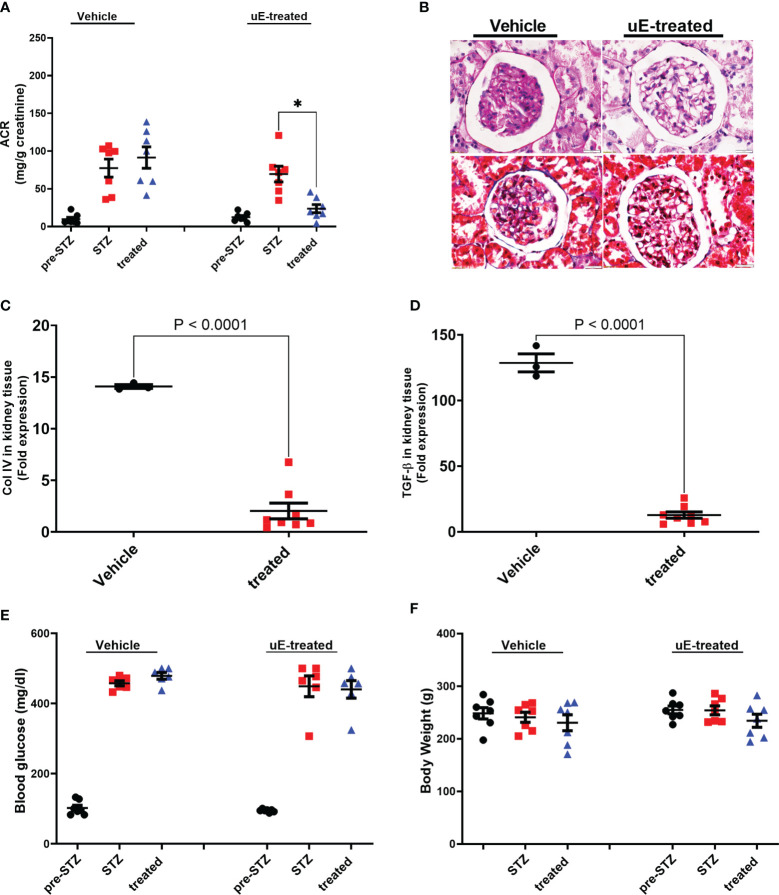
Renal function and pathology DN treated with urinary exosomes. Scatter dot plot with line at mean with SEM showing; **(A)** average albumin-creatinine ratio in rats’ urine, collected before diabetes induction (pre-STZ), at 6-8^th^ week post diabetes (post-STZ), and after two weeks of vehicle or uE-treated (treated). *p< 0.05 was considered as significant by paired t-test. Panel **(B)** shows representative PAS and Masson Trichrome-stained images of DN rats’ kidney tissue sections after two weeks of vehicle or treatment (n=3/group). Scatter dot plot with line at mean with SEM showing; mRNA expression of Collagen IV **(C)** and TGF-beta **(D)** in kidney tissue; blood glucose **(E)** and body weight **(F)** in vehicle (n = 6) and uE-treated (n = 6-7). *p< 0.05 was considered as significant by unpaired t-test.

We next determined if uE from DN rats had similar upregulated miRs as found in uE of the DN patients. First, diabetes was induced in rats by STZ-injection and validated by significant rise in blood glucose (**Figure 5**). The rats developed kidney disease (Diabetic nephropathy, DN) as indicated by the rise in urine albumin-to-creatinine (ACR) ratio from 6^th^ weeks onwards after diabetes induction, relative their own baseline (before diabetic induction, **Figure 5A**).

We next determined the miRs upregulated in the exosomes from DN rats’ urine, collected between 6th-8th weeks, relative to baseline (before diabetes induction) using microarray. The analysis showed a rise in the levels of 28 miRs, including miR-24-3p and miR-200c-3p relative to baseline in DN rats, among this upregulated expression of 20 miRs were also observed in uE from DN patients (**Table 3**).

**Table 3:** Diabetic nephropathy associated miRs in rat and human uE.

		Rat uE	Human uE
S.No	Transcript ID	Fold Change (DN vs. baseline)	Fold Change (DN vs. DC)
1	miR-23b-3p	53.18	7.76
2	let-7d-5p	35.24	2.35
3	let-7c-5p	63.25	10.95
4	let-7e-5p	15.66	3.09
5	miR-26a-5p	17.87	10.09
6	miR-200b-3p	84.65	1.13
7	let-7a-5p	45.59	1.69
8	miR-200c-3p	26.79	40.43
9	miR-182	2.27	1.1
10	let-7b-5p	71.44	32.44
11	miR-30a-3p	9.67	1.44
12	miR-107	4.14	1.11
13	miR-10a-5p	2.86	1.02
14	miR-24-3p	8.69	5.87
15	miR-23a-3p	6.41	5.54
16	miR-194-5p	7.34	1.54
17	miR-378a-3p	2.21	3.13
18	miR-200b-5p	2.3	1.08
19	miR-92a-3p	2.14	7.45
20	miR-29a-3p	2.28	1.11

Twenty-eight miRs had significantly higher expression in the pooled uE from rats with diabetic nephropathy, collected between 6^th^ to 8^th^ of diabetes induction (DN), relative to their own baseline (before diabetes induction). The table list 20 miRs among these which were also found in uE from DN patients, relative to subjects with kidney disease. The microarray data was analyzed using TAC 4.0 analysis software.

### Diabetic rats treated with their own uE had attenuated renal pathology and higher expression of renoprotective miRs

To test the renoprotective potential *in vivo*, we injected an aliquot of the collected uE back into the DN rats. The uE treatment was given for two weeks (weeks 9^th^ and 10^th^ after diabetes induction). A group of DN rats were treated with the same amount of vehicle. Urine and kidney tissues were analyzed from these rats after two weeks of treatment. Relative to vehicle treated rats, uE treated rats had significantly reduced urine ACR in DN rats (**Figure 5A**). In addition, DN rats treated with uE displayed renal pathology that was milder as compared to vehicle treated rats, as indicated by (**Figure 5B**). Also, uE treated rats had reduced levels of Collagen IV and TGF-beta, relative to vehicle (**Figures 5C, D**). These fibrotic and inflammatory genes are also known targets for miR-200c-3p, and miR-24-3p. The vehicle treatment did not show any significant effects blood glucose and body weight on any of these parameters (**Figures 5E, F**).

**Figure 6:**
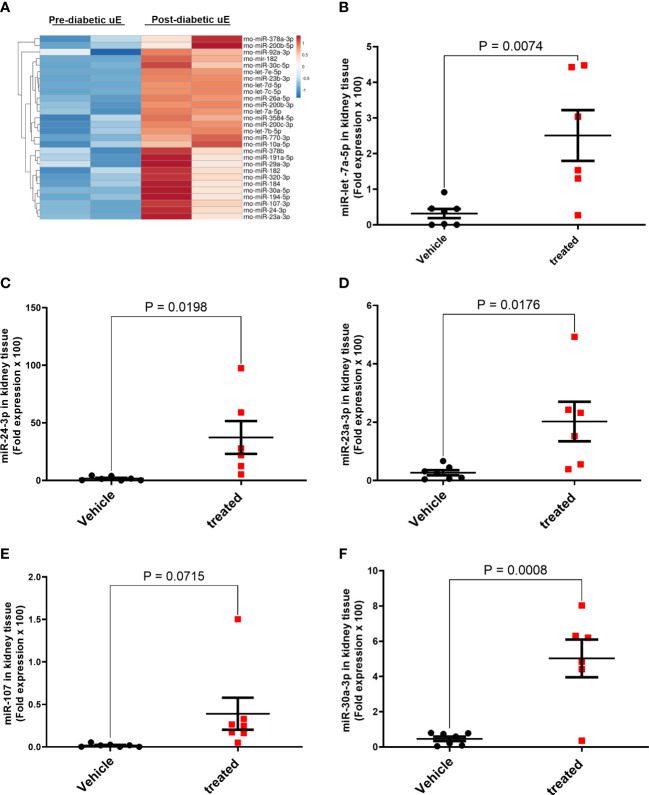
MicroRNA regulation in the kidney tissue of DN rats treated with urinary exosomes. Heat map **(A)** showing higher expression of 28 miRs in uE of DN rats, collected between 6^th^ to 8^th^ of diabetes induction, relative to their own baseline (before diabetes induction) using microarray. The microarray data was analyzed using TAC 4.0 analysis software. Scatter dot plot with line at mean with SEM showing fold expression of miRs, let-7a-5p **(B)**, miR-24-3p **(C)**, miR-23a-3p **(D)**, miR-107-3p **(E)**, and miR-30a-3p **(F)** in renal tissue uE-treated (treated), relative to vehicle treated (vehicle) DN rats (n=5-6/group).

We next tested if the uE treatment affected the expression of renoprotective miRs in DN rats’ kidneys by qPCR of 5 selected miRs that showed higher expression in uE from both DN patients and rats (**Table 3**; **Figure 6A**). The analysis showed higher expression of these 5 miRs in kidney tissue of DN rats treated with uE, relative to vehicle treated rats (**Figures 6B–F**)

## Discussion

Urinary exosomes (uE) from kidney disease patients exhibit a distinct biomolecular profile than healthy individuals (33–37). The distinct miRs profile of uE may indicate their selective sorting inside the exosomes corresponding to the pathophysiological condition (38, 39). However, it is unclear how the miRs regulation in uE and kidney tissue are related, as the relationship may shed light upon their role in renal pathogenesis. Regulation of miRs in uE has been widely studied for their potential to serve as renal disease biomarkers (33–35, 40), however, to the best of our knowledge, relationship between uE and renal regulation of miRs have not been studied in paired samples from humans.

In this study, we report a set of 15 miRs, including miR-24-3p, miR-200c-3p that had higher expression in uE but showed a decline in renal tissue in DN patients relative to controls without DN. The functional annotations of these 15 miRs suggest that their decline in renal tissue could promote disease progression. For example, miR-200c-3p has been suggested to regulate key biological processes in podocytes by maintaining intracellular calcium levels (41). Its role in cell invasion and migration has also been widely reported in renal cell carcinoma (42, 43). The miR-24-3p was found to regulate angiogenesis, wound healing, and fibrosis in diabetic nephropathy, along with its abundance in kidney diseases (44–46). Thus, the reduced renal levels of miR-24-3p in a renal biopsy from DN patients, found in our study, may further promote renal fibrosis and hence renal disease progression by targeting genes in VEGF and AGE-RAGE signaling pathways. Moreover, higher expression of these miRs in DN uE could thus explain their reduced renal levels, and thus, opposite miRs regulation between uE and kidney tissue may be of further significant. The strength of our study is that we have confirmed the opposite regulation of these miRs using paired uE and renal biopsy samples from DN patients.

Nevertheless, we further tested whether recouping the loss of such miRs may leverage kidney disease therapeutics. Since exosome are known to transfer their biomolecular content in the recipient cells (47), we tested whether recouping the lost miRs in kidney tissue by injecting them back via uE injection could attenuate pathology. We found that uE treated rats had attenuated renal pathology and recovered expression of miRs including such as let-7a-5p, miR-30a-3p, miR-23a-3p, and miR-24-3p. The injected uE expresses high expression of 20 out of 28 DN-associated miRs identified in humans including, miR-200c-3p, miR-24-3p, let-7a-5p, miR-30a-3p, miR-23a-3p. We believe that the mechanism for attenuated pathology in uE treated rats is independent of glucose-lowering, as the uE treatment did not affect the blood glucose levels in DN rats. However, later transfer of renoprotective miRs may have a role. The expression of miR-let-7s family miRs were reported as antifibrotic miRs in diabetic kidney disease in humans (48, 49). Among the family, the circulating miR-let-7b-5p was found to be abundant in renal diseases (42, 43). Similarly, miR-30 has been shown ameliorate DN by targeting fibrotic genes, bats (50). Studies found uE miR-30a was highly expressed in T2DN patients (35) and miR-23a-3p inhibits the inflammatory response and fibrosis via targeting EGR1 in DN (51). Using STZ-rats, Liu et al., 2020 have also demonstrated a negative association of miR-24-3p with renal fibrosis progression in DN (44). Further analysis of miR-24b-3p revealed its abundant expression in human fibroblast tissue besides urine-derived exosomes. MicroRNA-24-3p has been shown to negatively regulate skeletal muscle fibrosis by targeting smad2 in the TGF-β signaling pathway, cardiac fibrosis by targeting fibroblast growth factor 11 (FGF11), and renal fibrosis by targeting FGF 11 (44, 52, 53). Moreover, the role of miR-24 in wound healing has also been suggested (46).

Our preliminary data based on the analysis of the pooled kidney tissues from vehicle and uE-treated DN rats suggested regulation of chemokine-cytokine pathways by uE treatment. However, further studies are warranted to confirm these preliminary findings.

Overall, using paired uE and renal biopsy samples from DN patients we demonstrated opposite regulation of miRs with reno-protective potential. These findings suggest that loss of miRs through exosomes may be associated with reduced renal levels, promoting renal fibrosis in diabetic nephropathy. We showed that compensating for the loss of such miRs in DN rats, by injecting back their urinary exosomes, improved their renal levels and attenuated renal pathology.

## Data availability statement

The data presented in this study are deposited in the GEO repository, accession number GSE225393.

## Ethics statement

The studies involving human participants were reviewed and approved by Institutional Ethics committee (IEC) of the Sanjay Gandhi Postgraduate Institute of Medical Sciences (IEC Code 2018-139-EMP-106). The patients/participants provided their written informed consent to participate in this study. The animal study was reviewed and approved by Institutional Animal Ethics Committee (IAEC) of Sanjay Gandhi Postgraduate Institute of Medical Sciences (Ref no. IAEC/P-21/25/2018). Written informed consent was obtained from the owners for the participation of their animals in this study.

## Author contributions

DM and BS performed the experiments, reviewed the literature, analyzed the data, and prepare the manuscript draft. RS performed the experiments and analyzed the data. PM reviewed the literature and *in silico* data mining. NP and SV provided renal biopsies and biological samples. ST designed the study, analyzed the data, and wrote and revised the manuscript. All authors reviewed the final manuscript draft. All authors contributed to the article.
