# Supplementary material for: Therapeutic potential of urine exosomes derived from rats with diabetic kidney disease
Source: Front Endocrinol (Lausanne). 2023 May 12;14:1157194. doi: 10.3389/fendo.2023.1157194 (PMC10213426; doi:10.3389/fendo.2023.1157194)
Supplement: Supplementary file 1 [file Table_1.docx]

**Supplementary Tables**

Table S1: Demographic details of enrolled participants from whom uE and renal biopsy samples were collected for Microarray study

|  | miR expressions in Renal tissues compared by microarray | | miR expression in urinary Exosomes compared by microarray | |
| --- | --- | --- | --- | --- |
| Diagnosis | Diabetic nephropathy (DN) | Renal Calculus (Control) | Diabetic nephropathy (DN) | T2DM patients without DN (Controls) |
| Sample size (n) | 05 | 03 | 09 | 09 |
| Age (Yrs.) | 48±10 | 38±6.24 | 47.11±12 | 45.11±7.65 |
| Gender (Male/Female) | 5/1 | 2/1 | 6/3 | 6/3 |
| Duration of DM | 11±5.5 | 2.1±0.7 | 10±6.08 | 5.2±5 |
| BMI | 25.36±2.50 | 20±2.16 | 24±3.64 | 26±5.31 |

Table S2: Demographic details of enrolled participants from whom uE and renal biopsy samples were collected for qPCR studies.

|  | Urine and Renal tissue | | Urine |
| --- | --- | --- | --- |
| Diagnosis | Diabetic nephropathy (DN) | Renal Calculus (Control) | NDKD |
| Sample size (n) | 15 | 03 | 06 |
| Age (Yrs.) | 44±11 | 38±6.24 | 47±3.34 |
| Gender (Male/Female) | 8/7 | 2/1 | 4/2 |
| Duration of DM | 9±5.5 | 2.1±0.7 | 3.06±4.1 |
| BMI | 24.1±3.3 | 20±2.16 | 23±5.23 |
